# Endoscopic ultrasound diagnosis and management of mass-forming chronic pancreatitis with situs inversus

**DOI:** 10.1055/a-2849-6065

**Published:** 2026-05-05

**Authors:** Jiayi Ma, Ning Zhang, Wen Jiang, Zhendong Jin, Kaixuan Wang

**Affiliations:** 1 Department of GastroenterologyChanghai Hospital, Naval Medical University (Second Military Medical University)ShanghaiChina

**Fig. 1:**
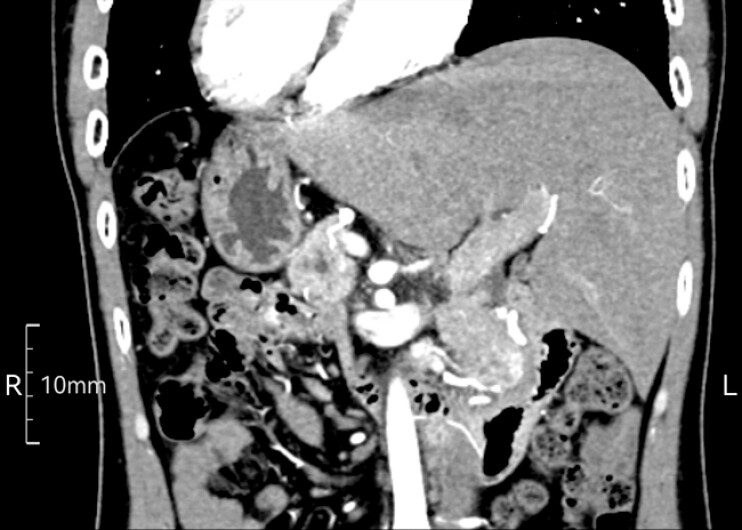
Pre-operative CT revealed heterogeneous enhancement of the pancreatic head, upstream pancreatic duct dilation and situs inversus. CT, computed tomography.

A 43-year-old man with recurrent acute pancreatitis (AP) over 4 years was admitted for etiological investigation. Situs inversus was incidentally detected upon computed tomography during a prior acute episode. Admission evaluations revealed a slightly elevated carbohydrate antigen level (200.34 U/mL), heterogeneous enhancement of the pancreatic head, and upstream pancreatic duct dilation ( Fig. 1 ).

**Fig. 2:**
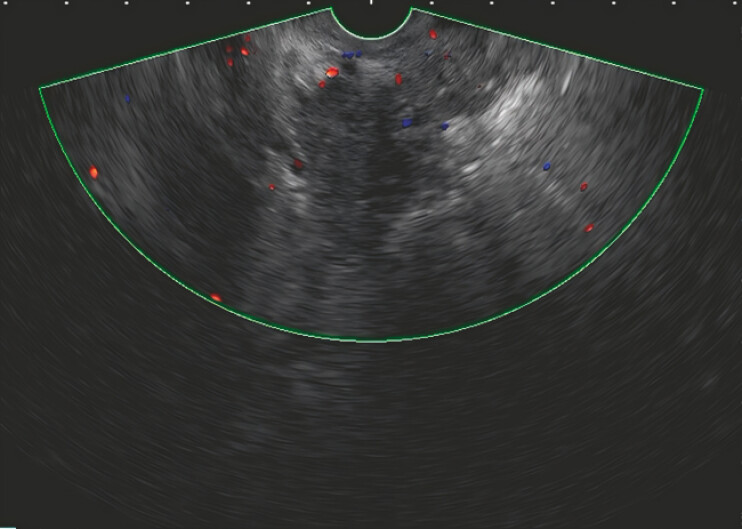
A hypoechoic lesion was identified in the pancreatic head, yet the pancreatic parenchymal texture remained intact.

**Fig. 3:**
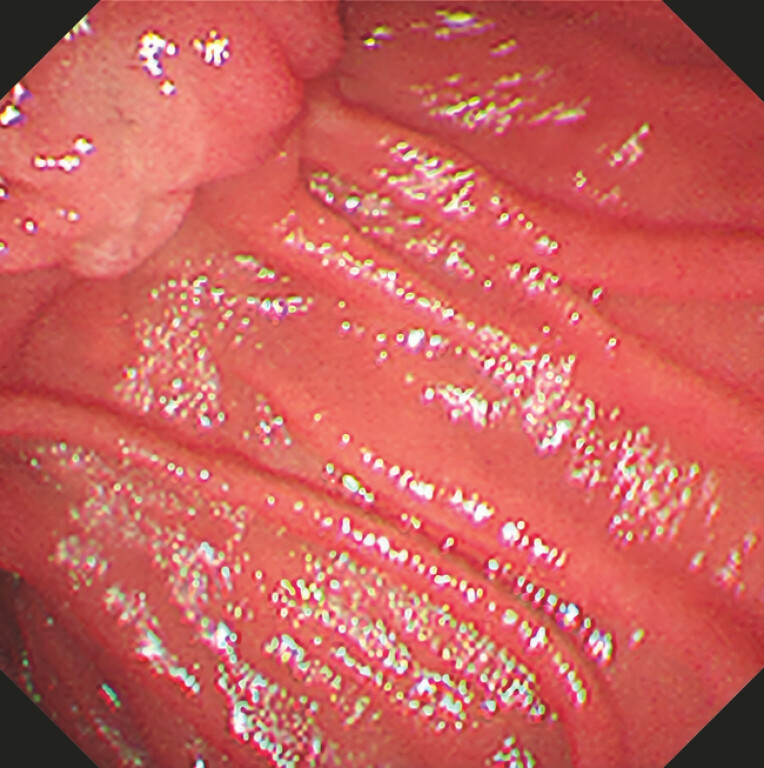
Deep cannulation failed due to the awkward papilla position.

**Fig. 4:**
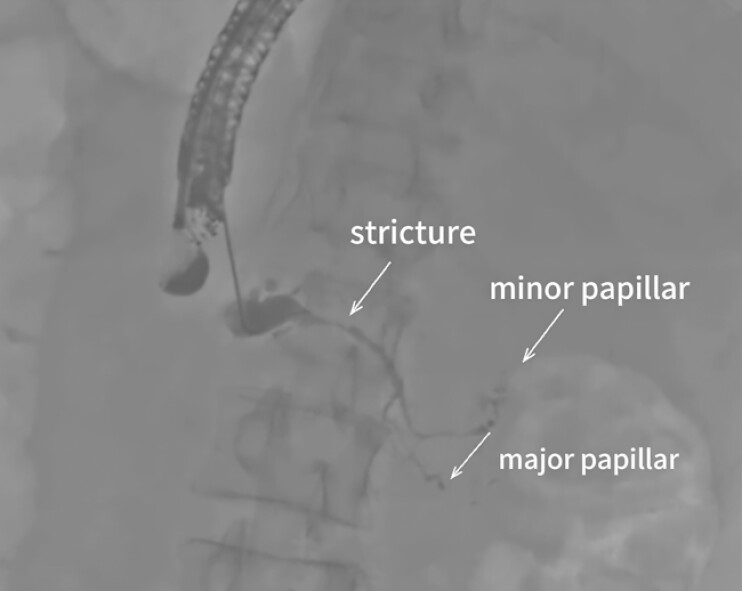
Fluoroscopy showed the stricture in the pancreatic duct at the pancreatic neck.

Endoscopic ultrasound (EUS) was technically challenging due to this anatomical variation, as it required opposite rotation directions and resulted in restricted operator range of motion. EUS showed a hypoechoic lesion in the pancreatic head and EUS-fine needle biopsy was performed ( Fig. 2 ). Pathological examination diagnosed mass-forming pancreatitis. For management, adequate drainage of the pancreatic duct will be crucial to alleviate his recurrent episodes. Endoscopic retrograde cholangiopancreatography (ERCP) was attempted first but failed due to the awkward papilla position ( Fig. 3 ). EUS-guided pancreatic duct drainage (EUS-PD) was alternatively performed. Except for the mirrored image, the procedure was consistent with routine practice. Pancreatography revealed a stricture and irregular contour of the main pancreatic duct, which was consistent with the Cambridge criteria for chronic pancreatitis ([0] Fig. 4 ). Guidewire passage via the major papilla was hindered by main pancreatic duct angulation; it was ultimately advanced via the minor papilla. The ‘ring’ technique rather than rendezvous was attempted so as to facilitate the subsequent stent exchanging [0]. However, the 7 F × 9 cm stent failed to pass the tight stricture even after a dilatation with a 4 × 4 cm dilatation balloon and was finally deployed bridging the stomach and pancreatic duct ( Video 1 ). No post-operative adverse event was observed. The follow-up will monitor the AP recurrence frequency and a repeat ring drainage attempt will be performed.

The 7F × 9 cm stent failed to pass the tight stricture even after a dilatation with a 4 × 4 cm dilatation balloon and was finally deployed bridging the stomach and pancreatic duct.Video 1

We present a challenging EUS-managed situs inversus case. Despite anatomical confusion and extreme scope rotation needed for diagnostic EUS, therapeutic EUS-PD was easily performed and superior to ERCP.

Endoscopy_UCTN_Code_TTT_1AS_2AI
